# Quick speech motor correction in the absence of auditory feedback

**DOI:** 10.3389/fnhum.2024.1399316

**Published:** 2024-06-06

**Authors:** Morgane Bourhis, Pascal Perrier, Christophe Savariaux, Takayuki Ito

**Affiliations:** Univ. Grenoble Alpes, CNRS, Grenoble-INP, GIPSA-Lab, Grenoble, France

**Keywords:** speech motor control, tongue afferents, speech perturbation, somatosensory feedback, compensatory response, noise masking

## Abstract

A quick correction mechanism of the tongue has been formerly experimentally observed in speech posture stabilization in response to a sudden tongue stretch perturbation. Given its relatively short latency (< 150 ms), the response could be driven by somatosensory feedback alone. The current study assessed this hypothesis by examining whether this response is induced in the absence of auditory feedback. We compared the response under two auditory conditions: with normal versus masked auditory feedback. Eleven participants were tested. They were asked to whisper the vowel /e/ for a few seconds. The tongue was stretched horizontally with step patterns of force (1 N during 1 s) using a robotic device. The articulatory positions were recorded using electromagnetic articulography simultaneously with the produced sound. The tongue perturbation was randomly and unpredictably applied in one-fifth of trials. The two auditory conditions were tested in random order. A quick compensatory response was induced in a similar way to the previous study. We found that the amplitudes of the compensatory responses were not significantly different between the two auditory conditions, either for the tongue displacement or for the produced sounds. These results suggest that the observed quick correction mechanism is primarily based on somatosensory feedback. This correction mechanism could be learned in such a way as to maintain the auditory goal on the sole basis of somatosensory feedback.

## 1 Introduction

Speech production can be assumed to be auditory in nature since the goal is to produce phonemic-relevant acoustic signals. This view is strongly supported by the huge difficulty hearing-impaired individuals have in learning to speak without hearing-aids (Gold, 1980; Kral et al., 2019). The importance of auditory inputs in speech motor control has also been demonstrated in the experimental paradigm of speech motor learning with altered auditory feedback. When speakers receive auditory feedback with an alteration of the phonemic-relevant acoustic characteristics, they adapt their speech according to this auditory alteration (Houde and Jordan, 1998; Jones and Munhall, 2005; Purcell and Munhall, 2006; Rochet-Capellan and Ostry, 2011). Somatosensory inputs which contains kinesthetic information are known to be important in human motor control. During speech production, speakers adapt to mechanical disturbances that affect somatosensory feedback even in situations in which the disturbance does not induce any auditory error in the speech sounds produced (Tremblay et al., 2003). It is thus important to know how somatosensory and auditory inputs interact in the speech production process.

In our previous study (Ito et al., 2020), the tongue showed a quick compensatory response when the tongue posture was suddenly disturbed by an external force during steady vowel production. The tongue was first pulled forward up to a maximum deviation, then moved back to compensate. This response movement had two phases. We considered the first phase to be the consequence of the passive elasticity of tongue tissue. In the second phase, the velocity of the backward movement increased. The latency of the onset of this second phase was about 130 ms after the onset of the perturbation. We considered the second phase of the response to tongue stretch to be the outcome of the influence of the neural sensory feedback. In this context, the crucial question was to clarify which sensory feedback – somatosensory or/and auditory—was involved in this phase of the response. An EMG study of tongue muscles involved in the control of the front part of the tongue, mainly the Anterior Genioglossus, was carried out on another set of participants for the same tongue stretch under similar experimental conditions (Ito et al., 2024). A significant increase in the EMG magnitude was observed there in response to a tongue stretch with a latency of around 50 ms. Computer simulations with a simplified linear mass-spring-damper model which included a delay between EMG signal and the force produced, showed that the latency of the EMG response (50 ms) is compatible with the latency of the onset of the second phase of the kinematic response. All these elements support our hypothesis that the observed kinematic response starting at 130 ms after the onset of the stretch could primarily be due to somatosensory feedback. However, given the latency of 130 ms, which has also been observed in perturbations of the auditory feedback (Xu et al., 2004; Cai et al., 2011), we cannot fully rule out a potential influence of the auditory feedback in the observed second phase of the response to tongue stretch.

To address this question, we examined whether the compensatory response of the tongue could be induced in the absence of auditory feedback. We carried out the test using the same tongue perturbation as in our previous study (Ito et al., 2020). To mask auditory feedback, a pink noise was presented during the speech production task. To maximize the effect of auditory masking, participants were asked to whisper the vowel and not to voice it. We compared the compensatory responses both in the articulatory and the acoustic domains between two auditory conditions, i.e., normal and masked auditory conditions. In line with our hypothesis of the predominant role of somatosensory feedback in the generation of the response to the tongue stretch perturbation, we expected that the compensatory response would be induced similarly and consistently regardless of auditory conditions. Two phases are thus expected in the response, and the latter phase, which is hypothesized to be driven by somatosensory feedback alone, is the focus of the data analysis.

## 2 Materials and methods

### 2.1 Participants

Twelve naïve young adults (5 females, 20–40 y.o.) participated in the experiment. They were all French native speakers and had no known speech or hearing impairments. They also had no history of profound injury that could impair somatosensation in the orofacial region. Participants signed the consent form approved by the local ethic committee, CERGA (Comité d’éthique pour la recherche, Grenoble-Alpes) (CERGA-AvisConsultatif-2021-18). One participant was excluded for not following the instructions. Eleven participants were included in the current analysis.

### 2.2 Movement and sound data acquisition

**Figure 1:**
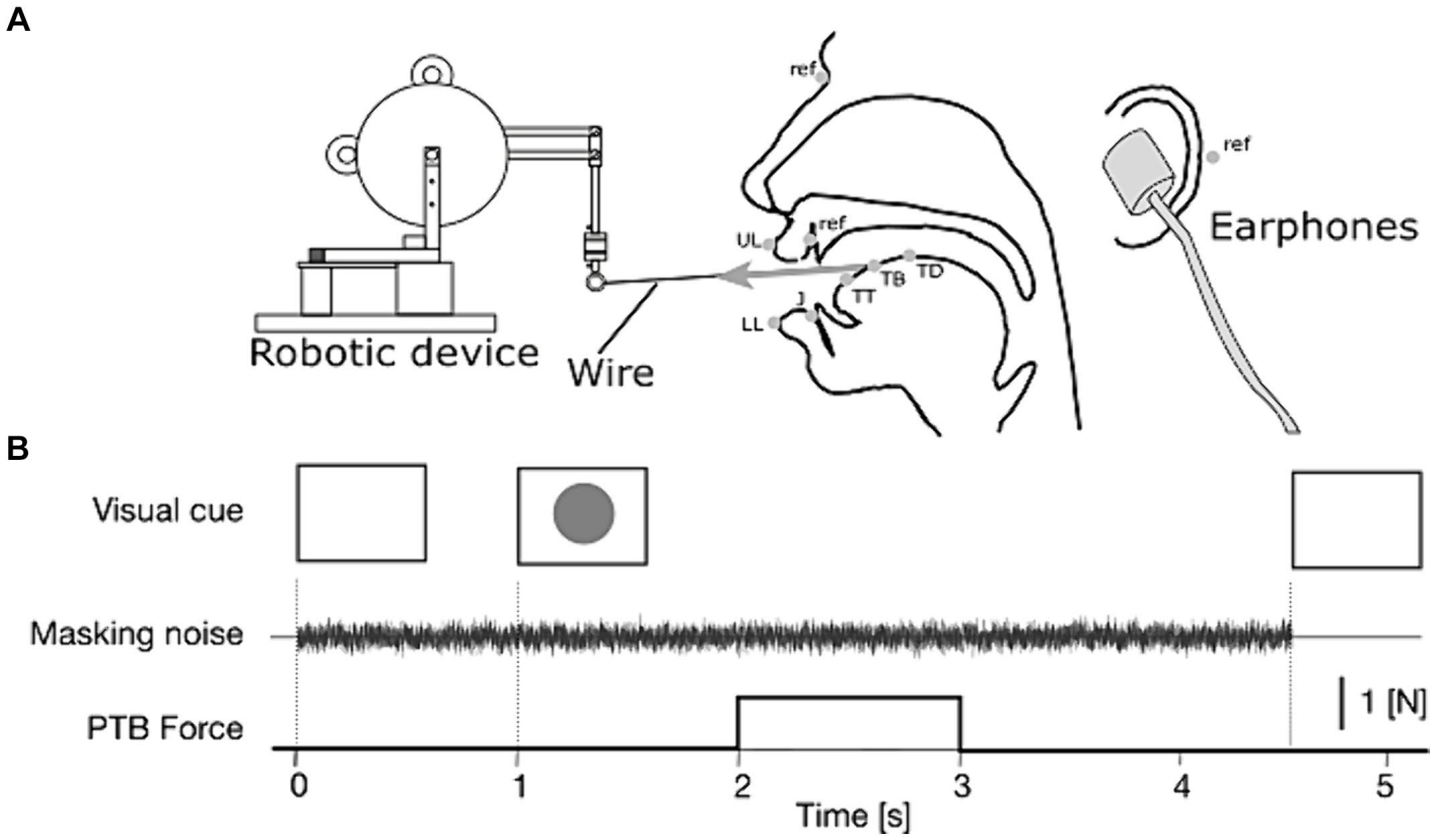
**(A)** Lateral view of the experimental setup (adapted from Ito et al., 2020). In the mid-sagittal view of the head (center of the panel), gray dots represent the positions of the EMA sensors: TT, tongue tip; TB, tongue blade; TD, tongue dorsum; J, jaw; UL, upper lip, and LL, lower lip and ref., reference markers on the nasion, left and right mastoids, and upper incisor. **(B)** Time sequence of each repetition of the speech task with tongue stretch perturbation (PTB) and auditory masking.

Electromagnetic Articulography (EMA, Wave, Northern Digital Inc.) was used to record articulatory movements in synchrony with the recording of the produced sounds. For the production of a front vowel such as /e/, the articulatory movement is primarily characterized by the tongue position in the mid-sagittal plane. Hence, EMA sensors were attached to the tongue in the mid-sagittal plane (Figure 1A): tongue tip (TT), tongue blade (TB), and tongue dorsum (TD). We planned to set at 1 cm the distance between two neighbor sensors on the tongue. However, this target distance was slightly adjusted in each participant depending on the size of the tongue. Finally, the resultant distances were 1.1 ± 0.68 cm between TT and TB, and 1.1 ± 0.85 cm between TB and TD. Additional sensors were attached to the upper and lower lips (UL and LL), and to the jaw (J), to record potential movements of articulators other than the tongue, which might affect vowel acoustics after the application of tongue stretch. For head movements’ correction in the off-line analysis, four reference sensors were attached to the upper incisors in the mid-sagittal plane, and the nasion and the left and right mastoids. The participant’s head was held in place with a head holder. After each recording, the palate contour in the mid-sagittal plane was recorded by tracing the surface of the palate using an EMA sensor attached to the experimenter’s finger. The sensor data were recorded at a 200 Hz sampling rate, and the speech sound produced was recorded synchronously at a 22.05-kHz sampling rate.

### 2.3 Speech task and auditory masking

The speech task consisted of the sustained production of the whispered vowel /e/ for about 3.5 s. Vowel /e/ was selected on the basis of the results of our previous study (Ito et al., 2020), in which systematic and large compensatory responses to tongue stretch were observed for this vowel. Since our previous study also showed no reliable difference between voiced and whispered conditions, we selected a whispered production in order to increase the likelihood that the masking noise actually masks the auditory feedback. For this masking, we used a pink noise, which was presented through earphones (Natus Tip 300) at 80 dB SPL. In every trial, the speech task started with the mouth closed and ended by closing the mouth.

### 2.4 Tongue stretch perturbation

For the tongue stretch perturbation, we used the same experimental setup as in Ito et al. (2020) (Figure 1A). A small robotic device (Phantom Premium 1.0, Geomagic) was placed in front of the participant and connected to the tongue surface with a thin thread. The thread has two small anchors, which were attached to the tongue surface on both lateral sides of the tongue blade sensor (TB). The distance between each anchor and the TB sensor was set to be 1 cm on either side of the sensor. This target distance was adjusted in each participant depending on the size of the tongue. The tongue stretch was applied by pulling the tongue forward with a 1 N force for 1 s. The force was applied as a step function, with rise and fall phases of 5 ms to avoid mechanical noise in the robot.

### 2.5 Experimental procedure

The time sequence of each trial is represented in Figure 1B. Each trial was triggered manually by the experimenter after visually checking that the participants were ready with their mouths closed and with their throats cleared if necessary. The participants carried out the speech task in response to a visual cue (green circle) presented on a monitor. Under the auditory masking condition, the noise was presented from the end of the previous trial to accustom the participants to the noise before the speech task and then lasted to the end of the trial at hand. The onset of the tongue stretch perturbation occurred between 1 s and 1.5 s after the presentation of the visual cue that launched the speech task.

The recording was divided into three sessions. Each session included 5 voiced trials and 50 whispered trials. The participants were asked to speak the vowel /e/ aloud in the first 5 trials and to whisper it in the following 50 trials. The 5 voiced trials were used to make sure that the participants actually produced vowel /e/ (and not vowel /ɛ/ or /œ/). Hence, we did not apply any perturbation during these voiced productions. In the 50 whispered trials, both auditory conditions were tested (25 trials each) in randomized order. The tongue stretch perturbation was applied in a fifth of the pseudo-randomly selected trials, with the constraint that it never occurred in two consecutive trials and that it was applied in the same number of trials in both auditory conditions (5 trials each in one session). In total, 165 trials were carried out (150 repetitions of the whispered speech task and 15 trials with voicing). 15 perturbed trials were recorded in each auditory condition. Despite these precautions, one participant did not correctly sustain the vowel /e/ during the main task and was excluded from the analysis.

Before the main recording, we also carried out a practice session to ensure that the pronunciation of vowel /e/ was correct and not influenced by a regional accent. The participants practiced to whisper the vowel with and without the auditory masking. We also asked the participants to make sure that the level of masking noise actually did mask their whispered sounds properly.

### 2.6 Data preprocessing

We only analyzed the perturbed trials in the articulatory and acoustic domains. For each trial, time zero was aligned with the onset of the perturbation, and the analysis was applied to the time interval from 1 s before the perturbation onset to 1.5 s after the perturbation onset.

The articulatory movement data were first preprocessed by correcting the movement of the head with the reference sensors. Considering the inter-individual variability in articulatory positioning for vowel /e/ production, we evaluated the relative changes from baseline. Baseline was defined as the average position computed over the 50 ms preceding the perturbation onset, for each sensor and each participant. Since the recorded tongue movements include the influence of jaw movement, we subtracted the position of the jaw sensor (J) from the recorded positions of the tongue sensors (TT, TB and TD). Finally, an average movement signal was computed for all the perturbed trials and all the participants for each sensor and for each auditory condition.

For sound data, the first three formants (F1, F2 and F3) were extracted over the same time interval as for the articulatory data using LPC analysis (Rabiner and Schafer, 1978). In this extraction, the acoustic signals were first under sampled at 10 kHz in order to focus on the frequency range ([0, 5 kHz]) of the first four formants in adult speakers. Sliding time Hanning windows of 25 ms with a shift of 2 ms were used, and an LPC analysis of order 12 was carried out for each window. Four possible formant frequencies were thus extracted at a sample rate of 500 Hz, and the time variations of the first three formants were then computed on the basis of these four frequencies, using basic smoothness and continuity principles. As with the movement data, time zero was aligned with the perturbation onset; the variation of each formant over time was computed for each participant relative to baseline, which was computed as the average value over the 50 ms preceding the perturbation. Finally, an average variation over time was computed for all the perturbed trials and all the participants for each formant and for each auditory condition.

### 2.7 Data analysis

In line with our previous findings (Ito et al., 2020), we expected the response induced by the tongue stretch perturbation to consist of two phases according to the response latency. We interpreted the early phase as the result of the passive mechanical characteristics of tongue tissues, and the later phase as the response induced by neural sensory feedback. In the current study, we focused only on the later phase of response and examined whether the magnitude and timing of the response differ across the two auditory conditions.

**Figure 2:**
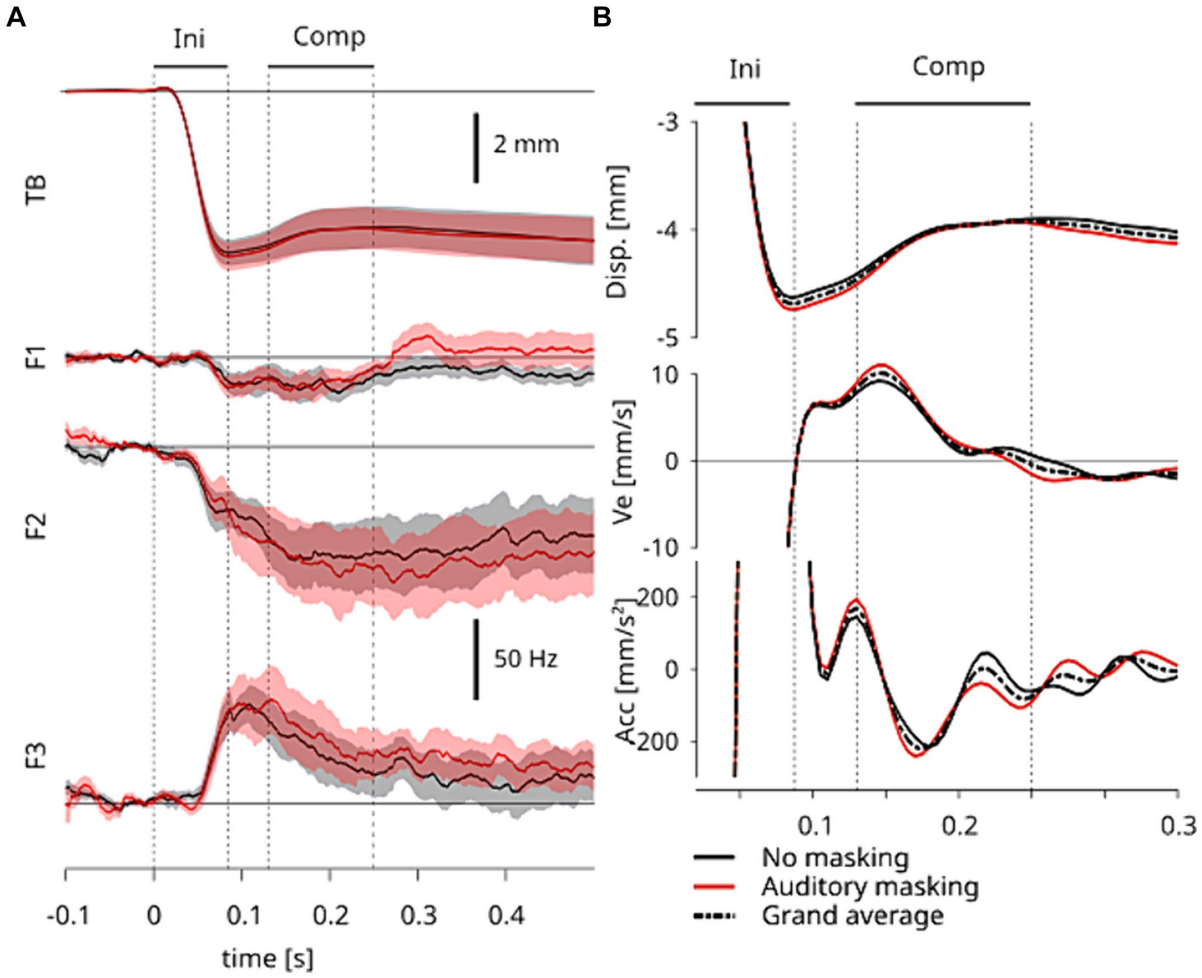
**(A)** Temporal pattern of the grand-averaged responses to tongue stretch perturbation. The top panel represents the horizontal displacement of TB sensor and the bottom three panels represent the first, second and third formants (F1, F2, and F3). The shaded area represents the standard error across participants. Time 0 corresponds to the onset of the perturbation. *Ini* represents the initial response directly induced by the perturbation and *Comp* represents the period of the compensatory response of interest induced by neural sensory feedback. The four vertical dotted lines represent the onsets and offsets in *Ini* and *Comp* periods. **(B)** Magnified views of the grand-averaged displacements of TB sensor (**A**, top panel), and their first (velocity) and second (acceleration) derivatives. The solid lines correspond to the data averaged within each of the two auditory conditions. The dashed lines represent the data averaged across two auditory conditions. The vertical dashed lines and the periods *Ini* and *Comp* are the same as in **A**.

Using the displacement, velocity and acceleration along the horizontal direction, we determined relevant time points of interest: the onset of displacement in response to tongue stretch; the time of the maximum displacement in response to tongue stretch; the onset and offset of the compensatory response induced by neural sensory feedback (See Results for the details). The horizontal displacements did not significantly differ between the two auditory conditions as shown in Figure 2B. So the data were averaged over the two auditory conditions and this grand-average was used to determine these time points. All the detected time points are represented by vertical dashed lines in Figure 2.

We measured the amplitudes in the sagittal plane of the initial displacement (from the onset of displacement to the time of maximum displacement, a period called “*Ini*” henceforth, Figure 2) and of displacement during the compensatory response (a period called “*Comp*” henceforth, Figure 2). For the sound data, we compared the formant frequencies F1, F2 and F3 at two time points: the time of maximal displacement in response to tongue stretch, and the offset of the compensatory response. For each measure, in the articulatory and acoustic domains, a one-way repeated measures ANOVA was applied to compare between two auditory conditions.

In line with our previous study (Ito et al., 2020), we expected the compensatory movement measured by the TB sensor in the mid-sagittal plane to take the shortest path to the original tongue contour, rather than returning to the exact original position before tongue stretch perturbation. We verified this behavior graphically by estimating the original tongue contour in the sagittal plane as the concatenation of two segments going from TT sensor to TB sensor and from TB sensor to TD sensor. To do this, we averaged the raw sensors’ positions over the 50 ms preceding the stretch onset.

We also assessed whether auditory masking affected the baseline articulatory posture for the production of the vowel /e/, bearing in mind that auditory masking might modify articulatory movement and posture – a phenomenon known as the Lombard effect (Luo et al., 2018). The typical behavior of the Lombard effect is that the energy of the produced sound increases, which is particularly marked in the energy of vowels, mostly without consciousness. We assessed possible consequences of this effect in terms of articulatory posture. We compared the baseline articulatory posture of the tongue including TT, TB and TD between two auditory conditions using repeated measures ANOVA.

## 3 Results

### 3.1 Assessing possible influence of Lombard effect on articulatory postures

**Figure 3:**
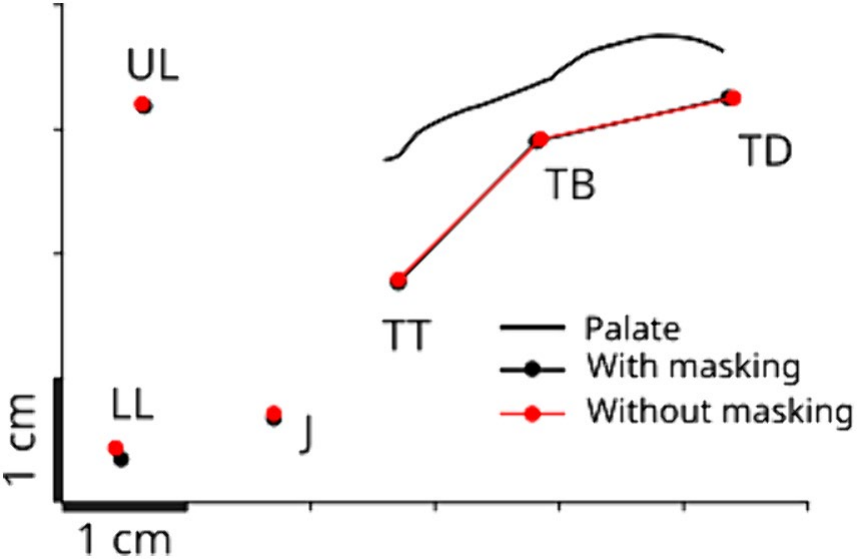
Baseline articulatory posture during the production of vowel /e/ for a representative participant in the mid-sagittal plane. The posture is obtained by taking the average across the 50 ms before the perturbation onset and across all the perturbed trials. The black line represents the recorded palate contour. TT, TB, and TD correspond to the tongue tip, blade and dorsum sensors, UL and LL, to the upper and lower lips sensors, and J, to the jaw sensor (see also Figure 1).

We first verified whether the auditory masking affected the baseline articulatory posture for the production of the task utterance in our experiment. We compared the positions of the three tongue sensors (TT, TB and TD) in both auditory conditions. Figure 3 shows a representative example obtained for one participant, which is the baseline articulatory positions in the two auditory conditions. Repeated-measures ANOVA showed no reliable difference between the two auditory conditions (*F*(1,50) = 0.025, *p* > 0.8), and no significant interaction effect between sensors and auditory conditions (*F*(2,50) = 0.016 *p* > 0.9). This indicates that auditory masking did not affect the basic achievement of the articulatory position for vowel /e/.

### 3.2 Compensatory responses to tongue stretch perturbation

Figure 2A shows the horizontal displacement of the TB sensor (top panel) and the corresponding F1, F2 and F3 changes (bottom three panels) observed in response to tongue stretch over time. As in our previous study (Ito et al., 2020), the compensatory response had consequences in the articulatory and acoustic domains alike.

The tongue stretch perturbation first induced a fast forward displacement of the tongue, as characterized by the TB sensor, up to a maximum (first period, called “*Ini*,” in Figure 2). In a second period of the response, the amplitude of displacement decreased as the result of the combination of passive and compensatory effects. As in our previous study (Ito et al., 2020), we identified three time points of interest to characterize these two periods in the response. The time of *maximum displacement* characterizes the end of the first period, which is purely due to passive effects. For the second period we relied on the velocity and acceleration profiles, and identified a compensatory response that we consider to be induced by neural sensory feedback. Figure 2B shows a magnified view of the displacement, velocity and acceleration of the TB sensor. We observed an increase in the velocity, which we characterized in time by *the peak of acceleration*, which corresponds to an inflection point in the displacement. This second time point marks the onset of the compensatory response due to neural sensory feedback. The offset of this compensatory response is characterized by the subsequent *velocity zero-crossing*, which is the third time point of interest. These points are indicated in both panels of Figure 2 by three vertical dashed lines points of interest. The compensatory response due to neural sensory feedback (“*Comp*”) is the focus of our analysis.

When the articulatory responses in the two auditory conditions were compared, we observed that the averaged values are mostly similar across participants in all variables. This can be seen in the top panel of Figure 2A, where the shaded areas represent standard-errors across participants for both auditory conditions. The clear overlap of the shaded areas between auditory conditions suggests no significant difference specifically in the *Ini* and *Comp* periods. This is quantitatively assessed below with repeated measures ANOVA.

**Figure 4:**
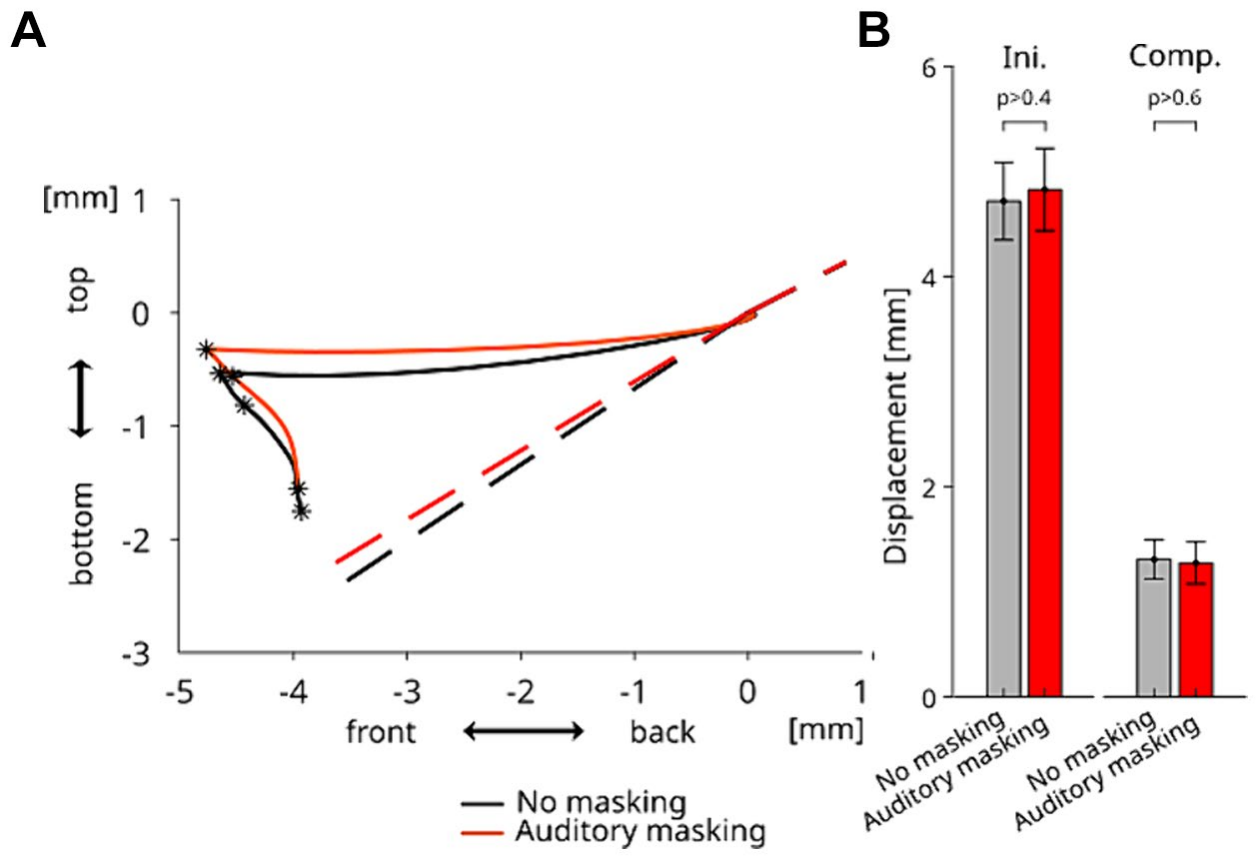
**(A)** Grand-averaged displacements of TB sensor in the mid-sagittal plane from the onset of the perturbation (Time 0) to the offset of the compensatory response induced by neural sensory feedback, for each auditory condition. The dashed lines represent the estimated original tongue contours in each auditory condition. **(B)** Amplitude of the articulatory displacements of TB sensor during the *Ini* and *Comp* periods in each auditory condition. Error bars represent the standard errors across the participants.

The averaged displacement of TB sensor in the mid-sagittal plane for each auditory condition—from the onset of the perturbation to the offset of the compensatory response induced by neural sensory feedback is presented in Figure 4A. As observed in our previous study (Ito et al., 2020), the tongue was first displaced horizontally in the forward direction due to the horizontal force applied to the tongue, and then the compensatory response occurred. Importantly, as in our previous study the compensatory movement did not follow the path back to the position before the tongue stretch perturbation. Instead it went in the downward direction so as to take the shortest path to a posture that preserved the original tongue contour (as estimated by the dotted lines in Figure 4A) in the alveo-palatal region, in which the constriction of the vocal tract occurs during the production of vowel /e/. In line with our observations in the horizontal direction, the trajectories in the mid-sagittal plane are similar for both auditory conditions. Figure 4B shows the amplitude of the movement response in the mid-sagittal plane for both periods *Ini* and *Comp*. The repeated measures ANOVA did not reveal any significant difference between the two auditory conditions in the amplitudes of both periods of the response (*F*(1,10) = 0.518 *p* > 0.4 for *Ini* and *F*(1,10) = 0.191 *p* > 0.6 for *Comp*). The results indicate that the auditory condition did not significantly affect the amplitudes of the initial changes and of the compensatory response.

The articulatory changes observed in response to the tongue stretch perturbation resulted in acoustical changes, as revealed in the variations of F1, F2 and F3 values. The rapid deflection of the tongue observed in the first period of the response induced a rapid decrease of F1 and F2, and a rapid increase of F3. The main effect associated with the decrease of the tongue deflection in the second period of the response is observed on F3 which clearly decreased and tended to return to its value before the perturbation onset. In F1, we essentially observe, for the same period of time, a stabilization with a slight non-significant trend to return to its original value, while F2 continued to decrease, but at a lower rate than in the first part of the response.

**Figure 5:**
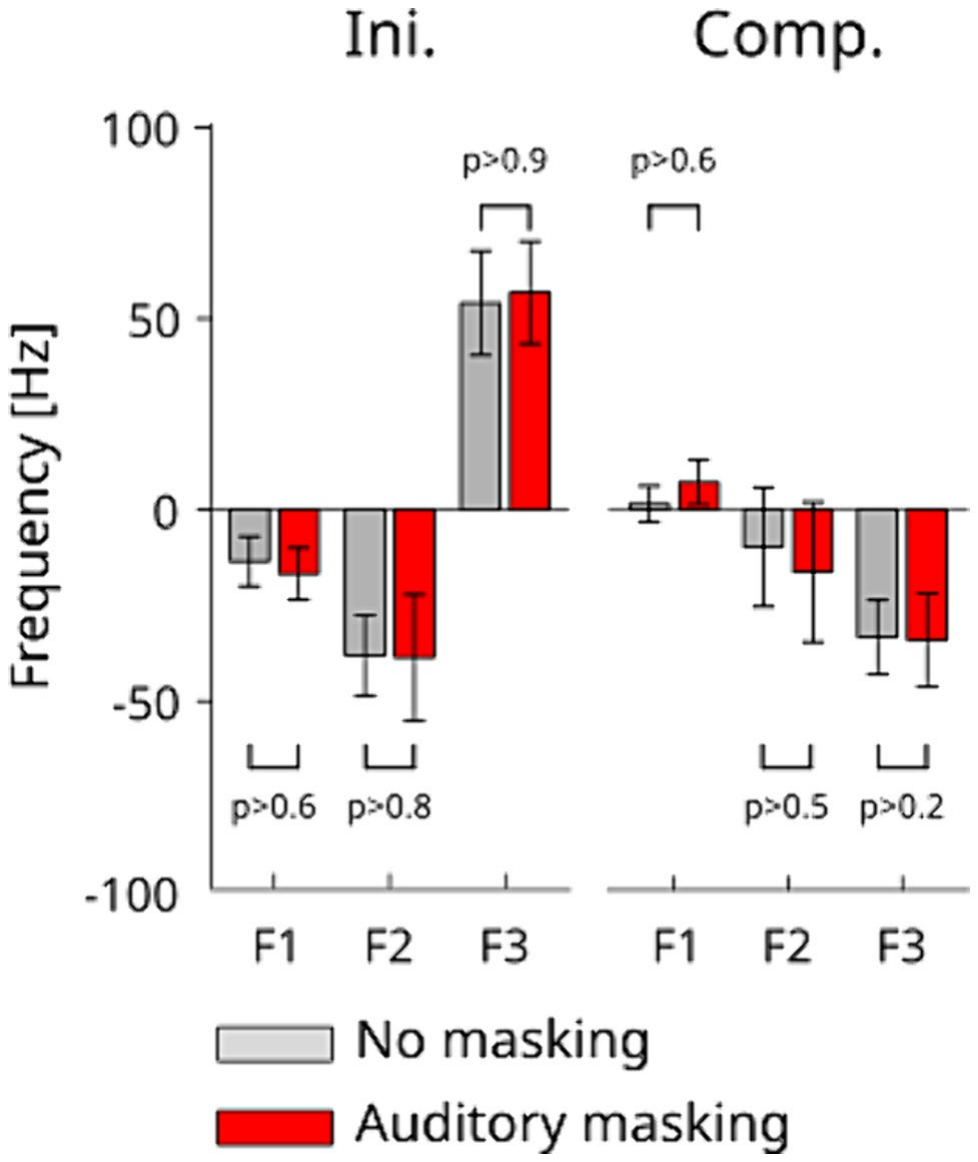
Amplitude of frequency change in F1, F2 and F3 during the *Ini* (left) and *Comp* (right) periods in each auditory condition. The error bars represent standard errors across the participants.

The averaged amplitudes of the normalized formant variations during the *Ini* and *Comp* periods together with the standard-errors are presented in Figure 5. At first glance, the figure confirms that significant formant changes were induced in the first period of the response to the perturbation (*Ini*), that only F3 shows significant compensation effects in both auditory conditions during the compensatory response due to neural sensory feedback (*Comp*), and that F1 does not show a clear trend toward a compensation for the effect of the perturbation. These observations are quantitatively confirmed by the statistical analysis: two-way repeated measures ANOVA show that only F3 underwent a significant change between the onset and offset of the compensatory response *Comp* [*F*(1,30) = 11.021 *p* < 0.003]. There was no interaction effect with the auditory condition for either formants [F1: *F*(1,30) = 0.358 *p* > 0.5, F2: *F*(1,30) = 0.088 *p* > 0.7, and F3: *F*(1,30) = 0.001 *p* > 0.9]. These changes were consistent in both auditory conditions. A one-way repeated measure ANOVA of the amplitude of formant variations during the *Ini* period showed no significant effect between the auditory conditions for each of the three formants [F1: *F*(1,10) = 0.276, *p* > 0.6 F2: *F*(1,10) = 0.021, *p* > 0.8, F3: *F*(1,10) = 0.006, *p* > 0.9]. Similarly, the two-way repeated measures ANOVA on the onset and offset times of the *Comp* period show no significant difference between the two auditory conditions in all three formants [F1: *F*(1,30) = 0.196, *p* > 0.6, F2: *F*(1,30) = 0.312, *p* > 0.5 and F3: *F*(1,30) = 1.395, *p* > 0.2]. The absence of difference between auditory conditions indicates that auditory masking did not affect the compensation in response to the tongue perturbation.

Note that a large increase of F1 was only observed after the *Comp* period in normal auditory conditions. Although this may possibly due to the compensation by auditory feedback, we did not pursue this further since this was beyond the current target hypothesis.

## 4 Discussion

Our main finding in the current study is that the response to the tongue stretch perturbation did not significantly differ between the normal and masked auditory conditions in both articulatory and acoustic domains. The results indicate that the current response is not dependent on auditory feedback and instead mainly relies on a somatosensory-basis. The compensatory mechanism driven by somatosensory inputs may be acquired through the development of speech production system, in order to integrate the cognitive requirements of speech communication. Auditory feedback is certainly important for speech production acquisition and development. However, the acoustic characteristics of speech sounds that are phonetically relevant for speech communication could be maintained in healthy adults by somatosensory inputs alone without using auditory inputs thanks to the learning of the association between somatosensory and auditory characteristics of speech sounds.

In the current experiment it is noteworthy that the participants faithfully replicated the quick compensatory response to the tongue stretch perturbation that we observed during vowel production in our previous study (Ito et al., 2020). More specifically we observed the two distinct phases of the compensatory response which we considered to be, respectively, mostly influenced by passive characteristics of the tongue and neural sensory feedback. We also confirmed another important finding of our previous study—when the tongue perturbation was applied in the forward direction during the vowel production, the response was induced to maintain the original tongue contour in the alveo-palatal region where the constriction of the vocal tract occurs, rather than to go back to the exact original position of the tongue.

Tongue stretch perturbation also changed the produced vowel sounds as evidenced in the F1, F2 and F3 variations. F3 was recovered as the result of the articulatory compensatory response, but not F1 and F2. This observation slightly differs from the results of our previous study, in which the compensatory effect was significant on both F1 and F3 (F2 variation was similar). In French vowel /e/ formant F1 (Helmholtz resonance of the resonator “back cavity + constriction” of the vocal tract) is mainly influenced by the change in the area of the constriction. F2 and F3 are mostly influenced by the position of the constriction along the antero-posterior direction (Apostol et al., 2004). F2 largely depends on the length of the back cavity of the vocal tract (half-wavelength resonance) which is quite large since /e/ is a front vowel, while F3 depends on the short length of the front cavity (quarter-wavelength resonance). Hence, the impact of the forward displacement of the tongue induced by the horizontal force applied by the robotic device can be interpreted as follows. In the majority of participants, the forward displacement narrows the tongue surface to the alveolar region of the palate, which reduces the constriction area and lowers F1. Also, the anteroposterior displacement of the constriction increases the length of the back cavity which lowers F2 and decreases the length of the front cavity, which in turn increases F3. Given the difference in the lengths of the front and back cavities, F3 is more sensitive to the front/back movement of the constriction (see nomograms in Fant, 1960). The effect of the articulatory change on F1 certainly varies among our participants depending on the shape of the alveolar part of the palate, which can be more or less curved along the front/back direction (see for example Brunner et al., 2009). Phonetically, for the French vowel /e/, a decrease in F1 associated with an increase in F3 endangers the correct perception of the vowel which could be identified as an /i/. In French, the spectral center of gravity between F2 and F3 is the most important cue to perceptually separate vowel /i/ from its neighbor vowels /e/ and /y/ (Schwartz and Escudier, 1987). The difference in F1 compensation between the current and the previous studies may be due to a difference in the effect of tongue-stretch perturbation. The change in F1 induced by the tongue perturbation in the current study is indeed smaller than the one observed with our previous study. This can be related to differences in the experimental setup and procedure. For the setup, the large interparticipant variability of the tongue structure prevents us to precisely control the sites of the recording sensors and anchors, and the direction of the tongue-stretch perturbation. For the procedure, the usage of whispered speech alone may affect the amplitude of initial change due to the tongue-stretch perturbation, because of differences in tongue postures. Different studies have indeed suggested that small differences exist in vocal tract configurations associated with the same vowels produced in whispered versus voiced condition (see for example Kallail and Emanuel, 1984). In addition, the whispered source, because it is a pressure source located at the back end of the vocal tract has been shown to excite the low resonance frequencies of the vocal tract with less efficiency than the voiced source (see Stevens, 1998). This can impact both the sensitivity of the participants to change in F1, which in turn alters their compensatory strategies, and the accuracy of the measure of F1 based on LPC. Hence our finding that the largest effect of the compensatory response is on F3 rather than on F2, which is less sensitive to front/back articulatory variations, or on F1, which is phonetically less critical, supports our idea that the compensatory response efficiently preserves the phonetically most relevant acoustic characteristics of the sound. This statement is all the more important since our current results show that this mechanism occurs without any influence of auditory feedback. It supports our hypothesis of a somatosensory based neural feedback mechanism tuned to preserve the auditory characteristics of the sound throughout the process of speech acquisition and development.

The use of auditory masking with a loud pink noise to cancel the influence of auditory feedback on the compensatory response could induce articulatory consequences due to the Lombard effect (Luo et al., 2018). This could have dramatically altered the articulatory strategies of the participants and thus the articulatory responses to the perturbation. However, this was clearly not the case in our experiment, since the comparison of the participants’ postures in normal and masked auditory conditions did not reveal any significant difference in the region of interest. The Lombard effect was negligible for articulatory movement in our participants.

The role of somatosensory inputs in speech production, independent of acoustic feedback, has been demonstrated in Patri et al. (2020), Tremblay et al. (2003) and Nasir and Ostry (2008). Jaw perturbation during speaking has led to somatosensory adaptation even in the absence of auditory error (Tremblay et al., 2003). Nasir and Ostry (2008) have also shown that cochlear implanted participants adapted to such a jaw perturbation even when their implants were turned off. These studies showed that somatosensory error was corrected in speech motor adaptation independently of auditory error. We also showed somatosensory based on-line feedback control here. Conversely to the above cited studies, the current tongue stretch perturbation was found to change both somatosensory and auditory feedback. These two errors are presumably compensated by the correction of somatosensory error alone. This suggests that the speech specific auditory goal can be achieved by somatosensory-based control.

This reliance on somatosensory feedback could be due to the benefit of shorter latency than auditory feedback which can take a longer neural loop. The current reflex was induced with the latency of 135 ms. Similar latency was found in auditory-based compensation in response to an auditory feedback perturbation. Cai et al. (2011) showed that, when formants were altered on line during the production of a sequence of vowels, auditory compensation was induced with a latency of around 160 ms. In addition, a study using pitch shift perturbation showed a latency of around 120 ms in the compensation during the production of disyllabic sequences (Xu et al., 2004) and of multi-syllabic nonsense words (Donath et al., 2002). However, the speech tasks used in those studies involved dynamic speech and were thus different from the task used in the current study, namely static speech consisting of sustaining a vowel for a few seconds. In cases involving similar static speech tasks, auditory compensations in response to altered auditory feedback were shown to involve longer latencies. Purcell and Munhall (2006) found a latency longer than 460 ms when the formant was perturbed during a sustained vowel production. Larson et al. (2000) also showed a latency longer than 200 ms in response to a pitch-shift perturbation occurring during the production of a steady “ah” sound. Hence, consistent experimental results suggest that in case of an on-line control of sustained vowel production, the contribution of auditory feedback can involve latencies longer than 200 ms after the perturbation. In this context, somatosensory compensation is important to correct earlier phases of speech sound.

The importance of auditory feedback in speech production is known. For individuals with congenital deafness, it is difficult (or mostly impossible) to learn to speak without being equipped with hearing devices. Post-lingually deafened individuals also show degradations in their speaking performance along the course of deafness’ evolution (Cowie et al., 1982). This has also been demonstrated in the experimental paradigm of speech motor adaptation with altered auditory feedback (Houde and Jordan, 1998; Purcell and Munhall, 2006; Guenther and Hickok, 2015). When a somatosensory perturbation is applied simultaneously with a perturbation of the auditory feedback, this key-role of the audition in speech motor control may be affected. Lametti et al. (2012) have shown in such an experiment that 21% of their participants did not compensate for the auditory perturbation and focused on the somatosensory one. On the other hand, Feng et al., 2011 found that error monitoring in the auditory domain plays a dominant role as compared to error monitoring in the somatosensory domain. Beyond these considerations, in the case of an on-line feedback control, as mentioned above, the latency of auditory compensation may be too long for efficient and stable control. Thus, it could be expected that auditory feedback plays a role in correction loops with a long delay, while stability of the control is ensured via another channel. This potential long latency contribution of the auditory feedback can be seen in the current dataset in which we observed different recoveries in F1 between normal and masked auditory conditions in a period later than 300 ms after the perturbation. Although we did not pursue this in the current study, both somatosensory and auditory feedback contribute to on-line control in the different periods. It would be interesting to clarify this point by altering on line the auditory feedback to increase or reduce the auditory error shift that is naturally induced by the tongue stretch perturbation.

## Data availability statement

Due to ethical agreement the data will be share upon request in context of a non disclosure agreement (takayuki.ito@grenoble-inp.fr).

## Ethics statement

The studies involving humans were approved by comité d’éthique pour la recherche Grenoble Alpes (CERGA). The studies were conducted in accordance with the local legislation and institutional requirements. The participants provided their written informed consent to participate in this study.

## Author contributions

MB: Writing – original draft, Writing – review & editing. PP: Writing – review & editing, Supervision. CS: Writing – review & editing. TI: Writing – review & editing, Funding acquisition, Supervision.
