# Supplementary material for: Quick speech motor correction in the absence of auditory feedback
Source: Front Hum Neurosci. 2024 Jun 6;18:1399316. doi: 10.3389/fnhum.2024.1399316 (PMC11187305; doi:10.3389/fnhum.2024.1399316)
Supplement: Supplementary file 1 [file Data_Sheet_1.PDF]

Supplementary Material

Quick Speech Motor Correction in the Absence of Auditory Feedback

Morgane Bourhis, Pascal Perrier, Christophe Savariaux, Takayuki Ito

*** Correspondence:** Morgane Bourhis: morgane.bourhis@grenoble-inp.fr

1.1 Supplementary Figure

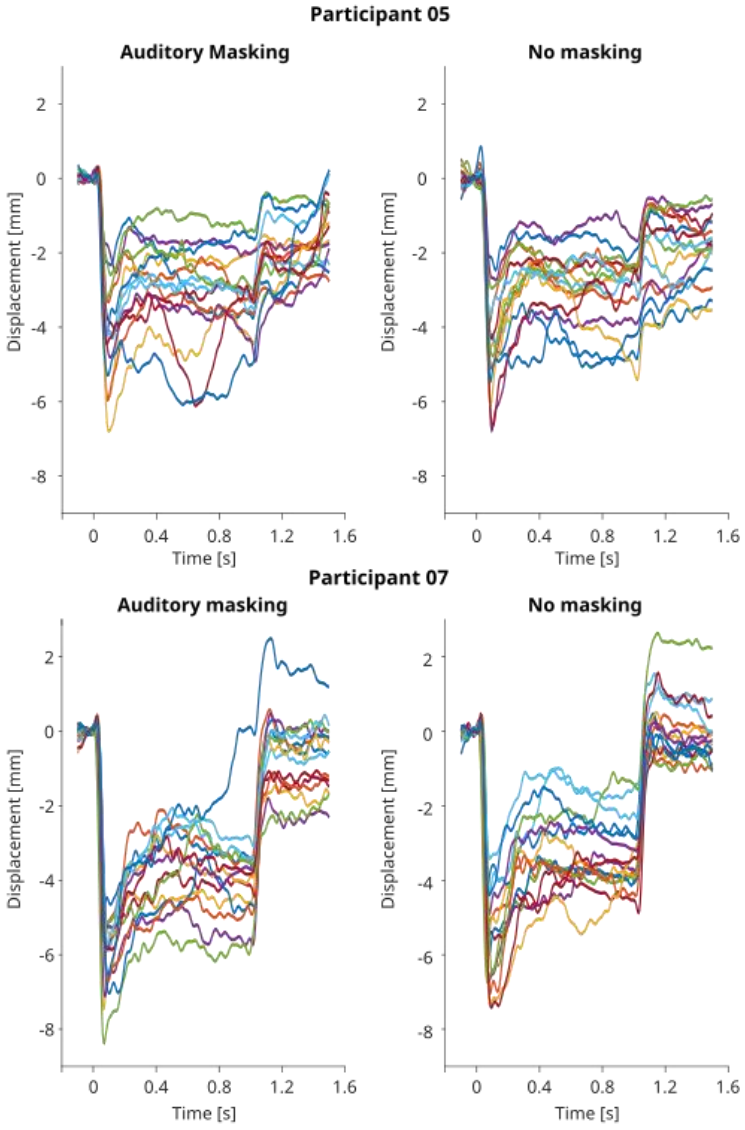

Supplementary Figure 1. Representative examples of horizontal displacement of the TB coil in each individual trial under auditory masking and no masking conditions
